# Clinical and Radiologic Outcomes of Augmented Partial Repair with Acellular Dermal Allograft and Superior Capsular Reconstruction in Massive Rotator Cuff Tears: 2-Year Follow-Up

**DOI:** 10.3390/jcm14010219

**Published:** 2025-01-02

**Authors:** Seung-Jin Yoo, Byung-Suk Kim, Ho-Hyup Kim, Sungwook Choi

**Affiliations:** Division of Shoulder Sports Medicine and Arthroplasty, Department of Orthopedic Surgery, Jeju National University Hospital, Jeju 63241, Republic of Korea; syoo06@gmail.com (S.-J.Y.); illseat88@gmail.com (B.-S.K.); hk728@naver.com (H.-H.K.)

**Keywords:** massive rotator cuff tears, acellular dermal allograft, augmented partial repair, superior capsular reconstruction

## Abstract

**Background/Objectives:** To evaluate the clinical and radiologic outcomes of arthroscopic augmented partial repair (APR) with acellular dermal matrix versus arthroscopic superior capsular reconstruction (SCR) in massive rotator cuff tears. **Methods:** The study included a total of 49 patients with massive rotator cuff tears who underwent arthroscopic APR (26 patients) and SCR (23 patients) between March 2018 and June 2021. Clinical scores, visual analog scores, and range of motion were collected preoperatively and postoperatively until the last follow-up. Preoperative and postoperative simple radiographs were evaluated for arthropathic changes and acromiohumeral distances (AHDs). Magnetic resonance imaging was performed to assess the integrity of repaired structures at 12 months postoperatively. **Results:** The average age of patients was 63.9 years (range 53–74 years), and the mean clinical follow-up period was 2.6 years (range 2.1–2.9). The average UCLA scores improved from 18.0 to 33.2 and from 16.3 to 32.1 in APR and SCR groups at the last follow-up, respectively. For the ranges of motion, the APR group consistently showed better external rotation ranges from the postoperative 6th month until the last follow-up (*p* < 0.05), and the APR group revealed better ranges of motion in forward flexion, abduction, and external rotation compared to the SCR group (*p* < 0.05). Postoperative AHD showed better improvement in the APR group than the SCR group (*p* < 0.05). Re-tears were found in two patients in each group (*p* > 0.05). **Conclusions:** Both APR and SCR groups showed comparable improvement in clinical outcomes in massive rotator cuff tears, while the APR group showed statistically significant improvement in the range of motion compared to the SCR group, especially for external rotations.

## 1. Introduction

Despite the recent advancement in arthroscopic surgical techniques and materials, massive rotator cuff tears are challenging to repair completely due to tendon retractions associated with fatty degeneration, loss of elasticity, and muscle atrophy [1]. Approximately 30% of all rotator cuff tears are still considered irreparable [2], and massive rotator cuff tears with advanced degeneration and chronicity have shown higher re-tear rates after an arthroscopic simple repair [3,4]. Currently, available surgical management for massive rotator cuff tears includes simple debridement with subacromial decompression, partial repair, augmented partial repair with various grafts, tendon transfers, muscle advancement, superior capsular reconstruction (SCR), and lastly, reverse shoulder arthroplasty [5,6].

In massive rotator cuff tears not amenable to complete repair, partial repairs (PRs) have been introduced for converting anatomically deficient rotator cuffs to functional rotator cuff tears by restoring the force couples of the deltoid muscle and remaining rotator cuff tendons [7,8]. However, PR is relatively vulnerable to higher structural failures, and outcomes for PR in massive rotator cuff tears vary, according to the previous literature [9,10]. High re-tear rates, reaching 40 to 90 percent after surgical repairs of massive rotator cuff tears, have implications of poor intrinsic healing potentials of chronically torn rotator cuff tendons [11,12]; however, advances in tissue engineering have developed acellular dermal allograft (ADA) by maintaining biomechanical and structural composition after de-cellularizing the dermis to potentially improve biological healing of repaired tendons [13]. Previous studies have reported improved clinical outcomes after augmented rotator cuff repair using ADA compared to primary repair in massive rotator cuff tears without significant complications [14]. In addition, a biomechanical cadaveric study showed that ADA augmentation has significantly decreased the superior translation of the humeral head [15]. SCR is another surgical option for massive rotator cuff tears that are simply irreparable by purportedly providing superior structural stability to the glenohumeral joint as a static stabilizer and reducing subacromial contact pressures [15]. Among many available graft options, the use of ADA is free from donor-site morbidity in autografts [16] and the risk of immune response in xenografts [17]. Previous studies have reported improved clinical outcomes with biologic incorporation of the grafts and no progression of glenohumeral arthritis after SCR procedures with ADA [18,19,20,21]. Despite a number of previous studies with satisfactory outcomes, both augmented partial repair (APR) with ADA and SCR procedures have a paucity of long-term clinical data and outcomes, and the direct comparison between the two procedures has not been well-established.

The purpose of the current study was to evaluate the clinical and radiologic outcomes of arthroscopic APR with acellular dermal matrix versus arthroscopic SCR in massive rotator cuff tears. We hypothesized that APR with ADA would show better clinical improvement and lower re-tear rates compared to SCR.

## 2. Materials and Methods

### 2.1. Study Design

The current study is a retrospective study involving a total of 49 patients selected from 182 consecutive patients with massive rotator cuff tears, as evidenced by magnetic resonance imaging (MRI), from March 2018 to June 2021. Our retrospective study for data collection and study protocols was approved by the ethics board at the institution and carried out in accordance with the principles in the Declaration of Helsinki.

The inclusion criteria were as follows: (1) a symptomatic massive rotator cuff tears (tears encompassing greater than 5 cm (cm), according to Cofield classification [22], or tears involving two or more tendons) confirmed by arthroscopic exams, (2) patients aged between 40 and 90, (3) postoperative MRI performed at postoperative 1st year, and (4) a minimum clinical follow-up of 2 years. On the other hand, the exclusion criteria included: (1) a partial thickness or small to large-sized tears, (2) arthroscopic rotator cuff repair with biceps re-routing/augmentation, complete double-row repair, (3) arthroscopic revisional rotator cuff repair, (4) Hamada classification grade IV and V, (5) concomitant glenohumeral conditions (i.e., inflammatory arthritis, bipolar chondral lesions, recurrent shoulder dislocations), and (6) other concomitant conditions (i.e., deltoid dysfunction, axillary nerve palsy, and previous infection or trauma).

Among the included 49 patients, a total of 26 patients treated with APR with ADA were assigned to the APR group. PR was defined as remaining less than 50% of the uncovered footprint after the repair in the coronal plane with the restoration of the rotator cable in the sagittal plane without excessive tension on repaired tendons. The other 23 patients who were not amenable to even partial repair and treated with SCR with ADA were designated to the SCR group.

### 2.2. Clinical and Radiologic Assessment

All patients were clinically assessed using the University of California Los Angeles (UCLA) score, Constant–Murley score, and visual analog score (VAS) preoperatively, as well as at the postoperative 3rd, 6th, 12th, and 24th months. In addition, they were also evaluated in sitting positions with a goniometer for baseline ranges of motion in all four directions of the glenohumeral joints with forward flexion (FF), abduction (ABD), external rotation (ER) with the elbow at the side, and internal rotation (IR) (vertebral level reached by the thumb given in points) preoperatively and at each postoperative outpatient follow-up.

Each patient underwent preoperative imaging with simple shoulder radiographs and MRI within three months of the planned operation. All patients’ radiological findings and measurements were recorded by an independent reviewer, who is an orthopedic surgeon with a subspecialty in shoulder surgery, and was blinded to the study participants. Simple shoulder true anterior-posterior radiographs were evaluated for glenohumeral arthritic changes based on Hamada classification [23] and acromiohumeral distance (AHD) both pre- and post-operatively. Preoperative MRI was performed to evaluate the tear size in the coronal and sagittal planes, rotator cuff tear patterns based on the Collin classification [24], the tendon retractions based on the Patte classification [25], and fatty degeneration of the rotator cuff tendons based on the Goutallier classification [26]. Postoperative MRI was performed at the postoperative 12th month to assess the integrity of the ADA graft in SCR-ADA or PR-ADA complex, using the Sugaya classification [27,28]. Sugaya type IV and V were considered as re-tears. Since the SCR procedure does not involve the repair of the torn tendons, reconstructed ADA was evaluated to determine whether the integrity of the superior capsule was intact.

### 2.3. Surgical Procedure

All surgical procedures were performed by a single surgeon (C.S.W). Both APR and SCR procedures were performed arthroscopically in the lateral decubitus position with traction applied to the involved limb using a positioner (Spider, Smith, and Nephew, Watford, England, UK) under general anesthesia with a single-shot interscalene nerve block.

In all patients, a complete inspection of the glenohumeral joint was carried out through standard posterior viewing and anterior working portals to assess intraoperative tendon quality and other pathologies. Biceps tendons were performed with tenodesis with subsequent tenotomy. The tear size was measured using a graduated ruler for the medial-to-lateral length and anterior-to-posterior width. Reducibility of the torn cuff tendons to the original footprint at the tuberosity was assessed after the release of the adherent rotator cuff with bursectomy and before any procedure regarding biceps and rotator cuffs. In addition, acromioplasty was completed in all patients, and a thorough bursectomy was performed to gain proper visualization of subacromial space.

The ADA (CGDerm, CGBio Co., Dae-Woong Pharm, Seoul, Republic of Korea) used in APR and SCR procedures was freeze-dried, cut-to-size, and packaged in a terminally sterilized double pouch and envelope. It was 4 cm wide and 7 cm long (surface area = 28 cm^2^) and 4 to 6 mm in thickness.

**Figure 1:**
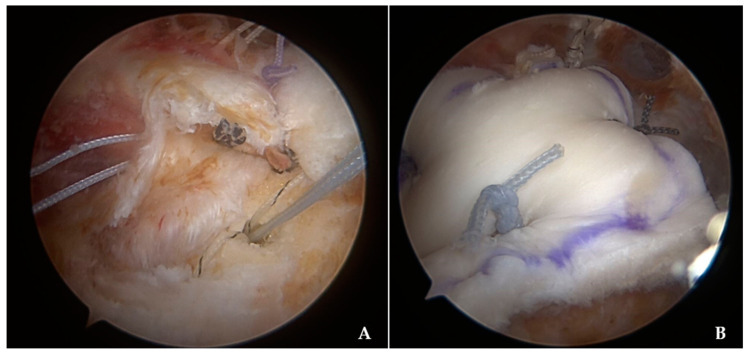
(**A**) Arthroscopic clinical image, as viewed from the lateral portal, showing partially repaired rotator cuff tendons with the medialized anchor positions and an additional suture anchor in the humeral head for the acellular dermal allograft fixation. (**B**) The acellular dermal allograft was applied on-lay to cover the footprint defect from the partially repaired rotator cuff tendon along with the side-to-side suture to the remaining cuff tendons for the firm fixation.

In the APR group, after adequate mobilization of retracted and torn cuff tendons, cuff mobility was assessed to ensure that the lateral margin of the torn tendons covering up to the footprint of the greater tuberosity without excessive tension. After the assessment of the reparability of the tendons and the preparation of footprint surfaces, medial row anchors (Bio-Corkscrew suture anchor; Arthrex, Naples, FL, USA) were inserted into the medial edge of the footprint for partial coverage. The medial edge of the footprint was prepared just lateral to the articular margin of the humeral head. Either a scorpion (Arthrex, Naples, FL, USA) or suture hook (Linvatec, Largo, FL, USA) was used to pass the sutures through tendons near the musculotendinous junction. Even though most cases used two medial anchors, the number of anchors used for the partial repair depended on the size and pattern of the tears. With the medialization of the footprint and adequate mobility of rotator cuffs, the partial repair was able to cover up not only the medial-to-lateral but also the anterior-to-posterior aspects. After the partial repair, the size of the uncovered footprint was measured with the graduated ruler, and ADA was tailored to be approximately 0.5 cm larger than the measured defect at each border. The remaining anchor threads on the anterior and posterior sides after the partial repair were not cut after the repair and were used later for the proximal fixation of the ADA (Figure 1A). ADA was placed on-lay over the partially repaired cuff tendon and firmly fixated with side-to-side sutures to the remaining partially repaired rotator cuff tendons with the arm in a neutral position (Figure 1B).

**Figure 2:**
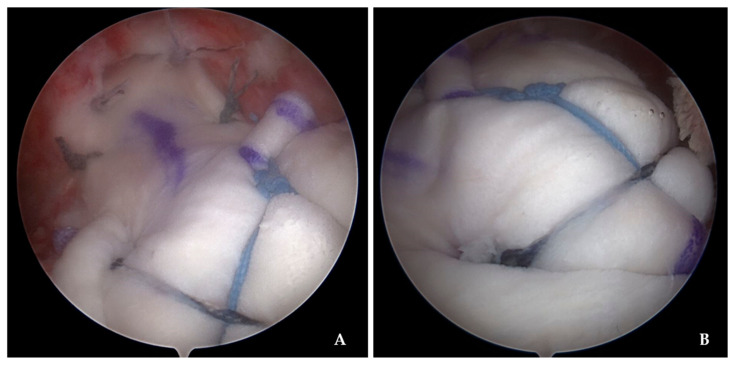
(**A**) Arthroscopic photograph, as viewed from the lateral portal, indicating the acellular dermal allograft fixation to the proximal glenoid and side-side suture to the remaining cuff tendons. (**B**) Arthroscopic image, as viewed from the posterolateral portal, showing the distal end of the acellular dermal allograft was firmly fixated to the humeral head with additional double-row fixation with the lateral anchors.

In the SCR group, even after the mobilization of the torn tendons, when the tendon retraction and mobility were not adequate for footprint coverage and reparability, SCR procedures were considered and performed. The superior aspects of the glenoid and bone beds of the greater tuberosity were prepared with a shaver and a motorized burr. Two anchors were placed approximately 0.5 cm medial to the superior labrum, which is considered the normal origin of the superior capsule and is the site for the proximal fixation of the ADA. Another double-threaded suture anchor was inserted in the footprint of the greater tuberosity, which is the site for the center fixation of the ADA. After retrieving sutures from the three anchors through the lateral portal, sutures from the subacromial spaces were passed through their respective holes in the ADA. Specifically, the suture limbs from each of the glenoid anchors were passed 2 mm anterior and 2 mm posterior to their respective marks on the graft with an antegrade suture passer. The eyelets of the medial anchors were utilized as pulleys to deliver the graft into the subacromial space. After the medial placement of the graft, the two untied suture limbs were tied to each other as a static knot in the subacromial space. The suture limbs from the greater tuberosity were tied in the center of the ADA for firmer attachment to the footprint. The anterior and posterior margins of the ADA were sutured side-to-side to subscapularis anteriorly and to infraspinatus posteriorly with additional suture threads, using either a scorpion (Arthrex, Naples, FL, USA) or suture hook (Linvatec, Largo, FL, USA) with the arm in a neutral position. After the fixation of ADA to the glenoid, humeral head, and remaining rotator cuff tendons, uncut glenoid suture limbs were utilized for additional double-row fixation of the ADA with two lateral anchors (Swivelock^®^ anchor; Arthrex, Naples, FL, USA) (Figure 2).

### 2.4. Postoperative Rehabilitation

The postoperative rehabilitation protocol was applied to all study participants in the same manner [29]. The abduction brace was applied until the initiation, the postoperative 6th week, of the continuous passive motion (CPM) exercises. The brace was briefly removed for physical therapy sessions and daily hygiene during the immobilization period for postoperative 6 weeks. Shoulder pendulum exercises, active ROM of elbow joint, active forearm supination and pronation, and active wrist and hand motions were all tolerated immediately after the surgery in both groups. Postoperative CPM exercises were initiated in the postoperative 6th week in four directions.. Muscle strengthening exercise was commenced after gaining full FF of the affected shoulder. All patients were educated and encouraged to perform daily home exercises by in-house physical therapists, and patients’ ROMs were serially evaluated at each postoperative outpatient follow-up. All patients were allowed to use the affected shoulder without restriction after 3 to 4 months postoperatively.

### 2.5. Statistical Analysis

The statistical analysis for the study was performed using the SPSS software version 24.0 (SPSS, Inc., Chicago, IL, USA). A Chi-square test was performed for comparison of categorical variables in demographics. Independent *t*-tests and paired *t*-tests were used to evaluate functional and radiologic outcomes between the groups, both preoperatively and postoperatively. A *p*-value < 0.05 was considered statistically significant. Primary outcome measures were the UCLA score, Constant–Murley score, VAS, and AHD; in addition, secondary outcome measures were any procedural complications.

## 3. Results

### 3.1. Patients

A total of 49 patients (32 male, 18 female) were enrolled and retrospectively reviewed. Baseline demographic data and preoperative clinical assessment are described in Table 1. All the study participants were monitored and followed up for at least 24 months (range: 25~35 months) postoperatively. There were 39 right and 10 left affected shoulders with the dominant arm involved in 39 cases (82.6%). Underlying diabetes mellitus was identified in a total of 37 patients,18 in the APR group and 19 in the SCR group.

### 3.2. Clinical Assessment

Preoperative clinical parameters of the UCLA, Constant–Murley, and VAS scores did not differ statistically between the two groups (*p* > 0.05) (Table 2). Clinical scores of UCLA and Constant–Murley did not differ statistically between the two groups throughout the two-year follow-up periods. However, VAS indicated better pain reduction in the APR group than the SCR group at the postoperative 12th month (*p* = 0.019).

In respect to the shoulder ROM, the APR group showed better improvement in ER with elbow at sides than the SCR group from the postoperative 6th month (*p* = 0.007) to the postoperative 24th month (*p*-value 0.002) with statistical significances. Furthermore, the APR group showed statistically significant improvement compared to the SCR group in FF (*p* = 0.025) and ABD (*p* = 0.002).

### 3.3. Radiologic Assessment

Preoperative and serial postoperative simple shoulder radiographs were evaluated for the changes in AHD and glenohumeral arthritis based on the Hamada classification. The APR group and the SCR group both showed increases in AHD. ADH improved by 5.3 mm to 9.9 mm and 4.6 mm to 6.3 mm in the postoperative 24th month in the APR and SCR group, respectively (Table 3). At the postoperative 24th month, the APR group had statistically significant increases in the AHD compared to the SCR group (*p =* 0.05). Hamada classification in both groups showed no significant differences between the two groups in the baseline (*p =* 0.351) and no significant changes in glenohumeral joint arthritis throughout the 2-year follow-up (*p* = 0.203).

The preoperative MRI assessment revealed that the majority of the rotator cuff tear patterns were type C and D based on the Collin classification, with 24 cases (88.5%) in the APR group and 16 cases (79.6%) in the SCR group. Fatty degeneration of each rotator cuff tendon, according to Goutallier classification, showed no statistically significant differences in each tendon (*p* > 0.05). The tendon retraction, according to the Patte classification, indicated grade 3 retraction shown in 20 cases (76.9%) in the APR group and 21 cases (91.3%) in the SCR group, but such a difference was not statistically significant (*p* = 0.254).

### 3.4. Complications

**Figure 3:**
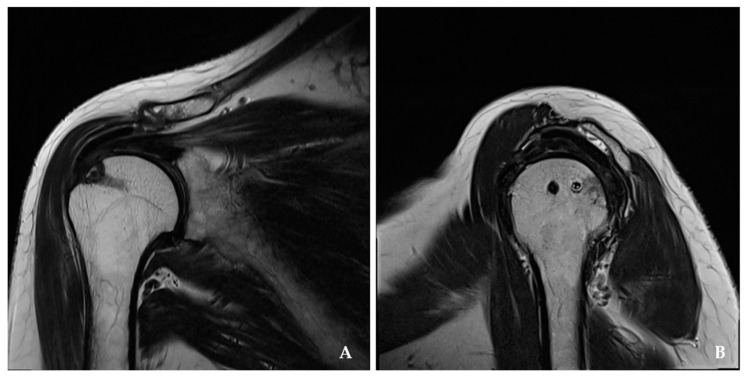
Augmented partial repair with the acellular dermal allograft was well preserved on-lay to the partially repaired rotator cuff tendons at the postoperative 1 year, and the medialized anchor position is notable. (**A**) magnetic resonance imaging of T1 coronal section. (**B**) magnetic resonance imaging of T1 sagittal section.

**Figure 4:**
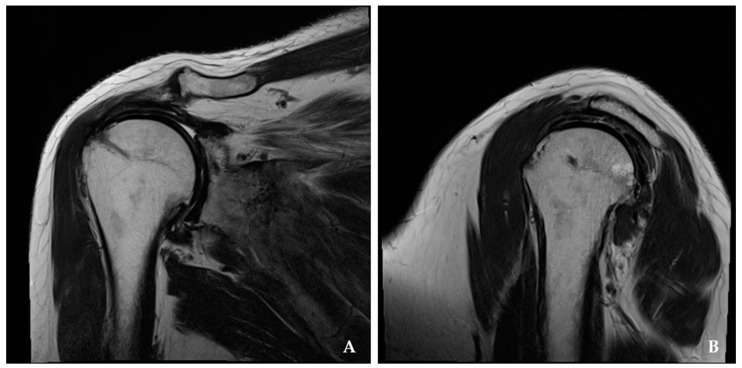
Superior capsular reconstruction with the acellular dermal allograft is shown at the postoperative 1 year. (**A**) magnetic resonance imaging of T1 coronal section. (**B**) magnetic resonance imaging of T1.

There were no known neurological or wound complications postoperatively. Postoperative MRI evaluated the integrity of the repaired tendon and reconstructed capsule in the APR and SCR groups, respectively (Figure 3 and Figure 4). There were two re-tears (7.6%) of Sugaya grade 4 and 5 in the APR group and two graft failure (8.6%) in the SCR group, but the re-tear rate did not show statistically significant differences on postoperative MRI (*p* > 0.05).

## 4. Discussion

In the current study, both APR with ADA and SCR in massive rotator cuff tears showed satisfactory improvement in clinical and radiologic outcomes with respect to the integrity of the repaired tendon, postoperative complication rates, and ADH. However, a notable finding from the current study was that the APR group exhibited slightly better postoperative ROM, especially in ER, and pain relief, which was apparent in the short term but then neutralized in the postoperative 2nd year, than the SCR group. Mild reduction in the postoperative range of motion after SCR has been frequently reported in the previous literature despite the improvement in functional outcomes [30]; however, relatively higher postoperative pain associated with SCR than APR with ADA in short-term is inconsistent with previous literature but is thought to be partly influenced by preoperative severe fatty infiltration of involved tendon and the relatively higher preoperative pain level in the SCR group [31].

There are immense challenges in treating irreparable, massive rotator cuff tears due to poor tendon quality, severe fatty degeneration of torn tendons, and limited mobility of retracted tendons [6]. The reparability of the massive rotator cuff tears cannot be accurately predicted before the actual intraoperative assessment of the rotator cuff pathologies. However, even if massive rotator cuff tears are intraoperatively considered reparable, the risks for re-rupture certainly depend on patients’ age, tear size, repair techniques, inappropriate postoperative rehabilitation, underlying diseases, such as diabetes mellitus and dyslipidemia, bone mineral density, smoking habits, and the fatty degeneration of the torn tendons [5,32,33]. With the rapid growth of the aging population with shoulder pain, patients older than 60 years of age comprise approximately 1/3 of rotator cuff tears, and the re-tear rate reaches up to approximately 45% in massive rotator cuff tears for older patients over 70 years of age [6,33,34]. When considering surgical repair for middle-aged to elderly patients with rotator cuff tears, it is essential to carefully consider not only cuff and glenohumeral joint pathologies but also chances of re-operations, such as revision repair or arthroplasty conversion surgeries. In addition, another factor contributing to the complexity in treating massive rotator cuff tears is that postoperative structural failure does not always result in clinical failure when force coupling is preserved [35].

A handful of surgical techniques have been introduced for treating massive rotator cuff tears; however, when full coverage cannot be achieved, graft augmentation can be a feasible alternative. Graft augmentations supplement the biological healing of repaired tendons and play a role as a spacer in subacromial spaces. Currently, available biologic scaffolds for graft augmentation comprise autografts, allografts, xenografts, and synthetic grafts [7,13,36]. Graft augmentation with autogenic or allogenic scaffolds was already introduced in the early 1980s; however, its usefulness has been brought back to attention with advances in tissue engineering [2]. A xenograft is no longer on the market due to its potential for immediate inflammatory responses, and autografts, such as tensor fascia lata, have the disadvantages of donor-site morbidity [14,36]. Although synthetic grafts have been reported to show favorable outcomes, available data and evidence are still limited [35,37,38,39].

In massive rotator cuff tears, intrinsic biological factors of torn tendons, such as hypovascularity and poor soft tissue qualities, contribute to higher re-tears and clinical failures [40]. The healing of rotator cuff tears is multifactorial, and the patient’s age and the degree of glenohumeral arthropathy are important factors to be carefully considered in surgical decision-making in terms of the degree of degeneration, reparability of the rotator cuff, and patient’s expectation in the postoperative level of sports or daily activities. In addition, as the re-tears are associated with multiple intrinsic and extrinsic factors, surgical failure has been reported to be biologic, rather than biomechanical [41]. The main purpose of ADA augmentation, in addition to partial repair without excessive tension, is to provide structural enhancement along with biological healing and cellular recovery for tendon regeneration. Furthermore, a previous animal study comparing primary repair versus APR with ADA in large rotator cuff tears showed that ADA augmentation resulted in superior histologic characteristics, including fibroblastic in-growth at the bone-to-tendon interfaces, neovascularization, and enhanced collagenous matrix formation [42]. Enhanced tendon-to-bone healing at the repair site is reported to be the greatest contributor to its low re-tear rate of 17%. In addition, medial tear at the musculotendinous junction is also important because a poor and degenerative tendon, as well as the chronicity of the tear, is frequently encountered in massive rotator cuff tears; consequently, appropriate tensioning of the repair construct, remaining vascularity of torn rotator cuffs, proper stress concentration at musculotendinous junction, and the use of bioaugmentation need to be carefully and critically considered preventing Cho type 2 failures [43,44,45]. The current study showed that the re-tear rate in the APR group was 2 out of 26 cases (7.6%). However, the recent literature on graft augmentation provided conflicting results. Mihata et al. showed no significant improvement in postoperative cuff integrity after patch augmentation compared to rotator cuff repair alone, with relatively high re-tear rates up to 62% [15]. On the contrary, a recent meta-analysis reported a reduction in re-tear rates and improved clinical outcomes, including better ROM as benefits of patch augmentation, the findings of which were well replicated in the current study as well [34].

Massive, irreparable rotator cuff tears also have a structural defect in the superior capsule, located on the inferior surface of rotator cuff tendons on the posterosuperior aspects [15]. Tears of the supraspinatus in a combination with infraspinatus tendons tears concomitantly sever the superior capsule [46], and structural deficits in the superior capsule may allow significant superior migration of the humeral head, aggravated in shoulder abduction positions, which could increase subacromial contact pressures and may expedite the degenerative process of rotator cuff arthropathy, if not reversed [47]. Mihata et al. first introduced SCR procedures using autologous tensor fascia lata graft and reported excellent short-term, as well as long-term, clinical outcomes with astonishingly low re-tear rates in irreparable, massive rotator cuff tears [46,48,49]. In addition, SCR using an ADA was also equally supported by favorable short-to-midterm improvements in clinical outcomes in a number of previous literature; however, more recent publications on SCR using an ADA have been challenged by inconsistent postoperative outcomes [19,50,51,52]. Differences in technical factors, patient factors, and precise indications for the procedure have not been fully defined, yet [50,53,54,55]. Muscle strength recovery after the SCR procedure was observed up to the postoperative 1 year [56], and the time required for minimal clinically significant changes after the SCR procedure in the natural course was reported to be about 1 year after the surgery [57]. However, after the first introduction of SCR by Mihata et al. [46], a recent systemic review reported that subsequent clinical studies showed re-tear rates to be approximately 19%, ranging from 0 to 47.6% and that most graft failures in SCR procedures occurred on the humeral side (69.8%), the glenoid side (13.2%), and the interstitial side (13.2%) [51]. In addition, among the middle-aged to elderly population with irreparable and massive rotator cuff tears, the SCR procedure showed equivalent clinical and functional improvement to reverse shoulder arthroplasty, especially in patients with Hamada grade 1 or 2 [58]. However, in a previous biomechanical study, when compared to the intact superior capsule, defects in the superior capsule showed increases in the external and internal rotation ROMs, as well as superior translation of the humeral head; consequently, the reconstruction of the capsule restored the stability of glenohumeral joints and functions as a spacer [55,59]. In a cadaveric study, SCR significantly decreased the rotational ROMs, which were abnormally increased preoperatively due to the superior capsule defect [60]. Post-SCR reduction in ROM was reported to be approximately 17% compared to the unaffected side, which was concordant with the outcomes of the current study [15].

In addition, the increase in the postoperative AHD was significantly greater in the APR group than the SCR group, owing to the normal shoulder anatomy where the rotator cuff sits on top of the superior capsule, providing greater downward compression force on the humeral head. A combination of the partially repaired rotator cuff and the ADA graft in the APR group naturally increases the spacer effect compared to the single layer of ADA-reconstructed superior capsule in the SCR group in the current study.

This study has a few limitations. First, the retrospective study design with relatively small sizes of study participants carries the possibilities for bias. Second, even though baseline demographics of both groups showed no significant differences in all variables, it is realistically difficult to predict whether the massive rotator cuff tears would be reparable and require an intraoperative assessment of the tendon qualities. Given that the APR and SCR procedures differ in surgical indications and principles of surgical techniques, it is quite challenging to direct hands-on comparison between the two groups.

## 5. Conclusions

Surgical and technological advances in the treatment of massive rotator cuff tears significantly improved clinical and radiologic outcomes. Our result indicated that both the APR group and the SCR group showed improved clinical outcomes; however, the APR group indicated better pain relief and ROMs, especially in external rotations, than the SCR group, which were consistently evident throughout the 2-year follow-up.

## Figures and Tables

**Table 1:** Patient demographic characteristics with the preoperative clinical and radiologic assessment.

	APR	SCR	*p*-Value
Gender			0.75
Male	18 (69.2%)	14 (60.9%)	
Female	8 (30.8%)	9 (39.1%)	
Age	64.3 ± 6.5	63.5 ± 6.0	0.67
BMI	25.3 ± 2.5	26.5 ± 4.7	0.26
Dominant hand			1.00
Right	25 (96.2%)	23 (100.0%)	
Left	1 (3.8%)	0 (0.0%)	
Operative side			1.00
Right	21 (80.8%)	18 (78.3%)	
Left	5 (19.2%)	5 (21.7%)	
Diabetes Mellitus			0.45
Yes	18 (69.2%)	19 (82.6%)	
No	8 (30.8%)	4 (17.4%)	
Smoking			0.44
Yes	23 (88.5%)	18 (78.3%)	
No	3 (11.5%)	5 (21.7%)	
ASA	2.0 [1.0; 2.0]	2.0 [2.0; 2.0]	0.54
VAS	6.0 [4.0; 8.0]	8.0 [6.0; 8.0]	0.08
UCLA	18.0 ± 4.2	16.3 ± 3.5	0.11
CONSTANT	46.8 ± 20.1	43.2 ± 15.4	0.51
FF, degree	170.0° [160.0; 180.0]	170.0° [155.0; 170.0]	0.42
ABD, degree	162.5° [90.0; 180.0]	160.0° [90.0; 170.0]	0.25
ER, degree	45.0° [40.0; 45.0]	45.0° [30.0; 45.0]	0.93
IR, point †	4.0 [3.0; 6.0]	4.0 [3.5; 6.0]	0.50
Involved tendons	3.0 [2.0; 3.0]	2.0 [2.0; 3.0]	0.59
Tear size	4.0 cm [3.0; 4.0]	4.0 cm [3.0; 4.0]	0.54
ML.	3.3 cm [2.6; 4.0]	3.8 cm ± 0.9	0.05
AP.	3.5 cm [3.0; 4.0]	4.0 cm [3.0; 4.8]	0.13
Goutallier Stages			
SSC	2.0 [1.0; 2.0]	1.0 [1.0; 2.5]	0.41
SST	3.0 [3.0; 4.0]	4.0 [3.0; 4.0]	0.16
IST	2.0 [2.0; 2.0]	2.0 [1.5; 3.0]	0.44
TM	1.0 [1.0; 1.0]	1.0 [1.0; 1.0]	0.75
Hamada Classification			0.20
Stage 1	33 (84.6%)	16 (69.7%)	
Stage 2	4 (15.4%)	5 (21.7%)	
Stage 3	0 (0.0%)	2 (8.7%)	
Stage 4 and 5	0 (0.0%)	0	
AHD	5.3 mm ± 1.4	4.6 mm ± 1.7	0.08

APR, Augmented partial repair; BMI, Body mass index; ASA, American Society of Anesthesiologists Classification; VAS, Visual analogue scale; UCLA, University of California Los Angeles; FF, Forward Flexion; ABD, Abduction; ER, External rotation; IR, Internal rotation; ML, Medial-to-lateral; AP, Anterior-to-posterior; SSC, Subscapularis; SST, Suprasupinatus; IST, Infrasupinatus; TM, Teres minor; AHD, Acromiohumeral distance; mm, millimeters. † Internal rotation was determined as the highest spine level that the patient’s thumb could reach in the back with 10 points, T7; 8 points, T7 to T12; 6 points, T12 to L3; 4 points, L3 to sacrum; 2 points, sacrum to greater trochanter; and 0 points, greater trochanter.

**Table 2:** Comparison in clinical outcomes between the augmented partial repair with the acellular dermal allograft and the superior capsular reconstruction with acellular dermal allograft throughout a 2-year follow-up.

	Patch (N = 26)	SCR (N = 23)	*p*-Value
**Postoperative 3rd month**			
VAS	3.5 [0.0; 5.0]	4.0 [1.0; 6.0]	0.60
UCLA	18.2 ± 5.1	22.8 ± 5.8	0.18
CONSTANT	35.0 ± 11.4	41.2 ± 16.2	0.49
FF	165.0 [150.0; 175.0]	160.0 [90.0; 165.0]	0.17
ABD	165.0 [140.0; 175.0]	150.0 [100.0; 165.0]	0.63
ER	42.9 ± 13.8	25.7 ± 21.3	0.09
IR	3.5 ± 1.8	4.6 ± 1.8	0.19
**Postoperative 6th month**			
VAS	1.0 [0.0; 4.0]	5.0 [1.0; 6.0]	0.06
UCLA	31.0 [29.0; 31.0]	28.5 [23.0; 31.0]	0.27
CONSTANT	58.0 [58.0; 68.0]	54.5 [38.5; 61.5]	0.43
FF, degree	175.0° [165.0; 180.0]	167.5° [165.0; 175.0]	0.08
ABD, degree	176.0° [117.5; 180.0]	165.0° [115.0; 172.5]	0.43
ER, degree	54.2° ± 26.9	21.7° ± 17.8	0.01
IR, point †	6.0 [4.0; 6.0]	5.0 [2.5; 6.0]	0.37
**Postoperative 1st year**			
VAS	0.4 ± 1.0	3.9 ± 3.2	0.02
UCLA	33.0 [29.0; 35.0]	29.0 [24.0; 32.0]	0.27
CONSTANT	66.5 [58.0; 71.0]	61.0 [45.5; 62.5]	0.18
FF, degree	177.5° [167.5; 180.0]	170.0° [160.0; 180.0]	0.27
ABD, degree	175.0° [165.0; 180.0]	170.0° [155.0; 175.0]	0.58
ER, degree	45.0° ± 11.5	28.3° ± 20.2	0.03
IR, point †	6.0 [6.0; 7.0]	6.0 [5.0; 6.0]	0.18
**Postopeartive 2nd year**			
VAS	2.0 [0.0; 2.0]	1.0 [0.0; 2.5]	0.89
UCLA	33.2 ± 1.6	32.1 ± 3.2	0.49
CONSTANT	71.7 ± 9.2	66.4 ± 6.4	0.25
FF, degree	170.0° [165.0; 180.0]	160.0° [150.0; 170.0]	0.02
ABD, degree	177.0° [167.5; 180.0]	160.0° [150.0; 165.0]	0.01
ER, degree	50.7° ± 10.2	27.5° ± 12.8	0.01
IR, point †	7.4 ± 2.5	6.3 ± 2.5	0.40

VAS, Visual analogue scale; UCLA, University of California Los Angeles; FF, Forward Flexion; ABD, Abduction; ER, External rotation; IR, Internal rotation. † Internal rotation was determined as the highest spine level that the patient’s thumb could reach in the back with 10 points, T7; 8 points, T7 to T12; 6 points, T12 to L3; 4 points, L3 to sacrum; 2 points, sacrum to greater trochanter; and 0 points, greater trochanter.

**Table 3:** Comparison in radiologic outcomes between the augmented partial repair with the acellular dermal allograft and the superior capsular reconstruction with acellular dermal allograft throughout a 2-year follow-up.

	Patch (N = 26)	SCR (N = 23)	*p*-Value
**AHD**			
Pre	5.3 mm ± 1.4	4.6 mm ± 1.7	0.08
POD3 M	9.3 mm ± 2.4	6.3 mm ± 1.8	0.01
POD6 M	9.7 mm ± 3.3	6.3 mm ± 4.7	0.28
POD1 Y	8.6 mm ± 1.9	7.0 mm ± 2.1	0.13
POD2 Y	9.9 mm ± 2.4	6.3 mm ± 2.9	0.05
**Hamada classification**			
Pre	1.0 ± 0.0	1.2 ± 0.7	0.35
POD3 M	1.1 ± 0.3	2.1 ± 1.4	0.04
POD6 M	1.2 ± 0.4	2.0 ± 1.4	0.55
POD1 Y	1.0 ± 0.0	1.8 ± 1.3	0.08
POD2 Y	1.0 ± 0.0	2.0 ± 1.7	0.20
**Collin classification**			0.15
Type A	1 (3.8%)	4 (17.4%)	
Type B	2 (7.7%)	1 (4.3%)	
Type C	13 (50.0%)	6 (26.1%)	
Type D	10 (38.5%)	10 (43.5%)	
Type E	0 (0.0%)	2 (8.7%)	
**Patte classification**			0.25
Grade 1	0	0	
Grade 2	6 (23.1%)	2 (8.7%)	
Grade 3	20 (76.9%)	21 (91.3%)	
**Sugaya classification**			
Grade 1–3 (Intact)	24 (92.3%)	21 (91.3%)	0.29
Grade 4–5 (Re-tear)	2 (6.7%)	2 (8.7%)	1.00

AHD, Acromiohumeral distance; Pre, Preoperative; POD, Postoperative day; M, Months; Y, Years; mm, millimeters.

## Data Availability

The data presented in this study are available on request from the corresponding author due to confidentiality matters.
